# Supplementary material for: Integrated Multi-Omics Profiling of Young Breast Cancer Patients Reveals a Correlation between Galactose Metabolism Pathway and Poor Disease-Free Survival
Source: Cancers (Basel). 2023 Sep 19;15(18):4637. doi: 10.3390/cancers15184637 (PMC10526161; doi:10.3390/cancers15184637)
Supplement: Supplementary file 1 [file cancers-15-04637-s001.zip › cancers-2564956-supplementary.pdf]

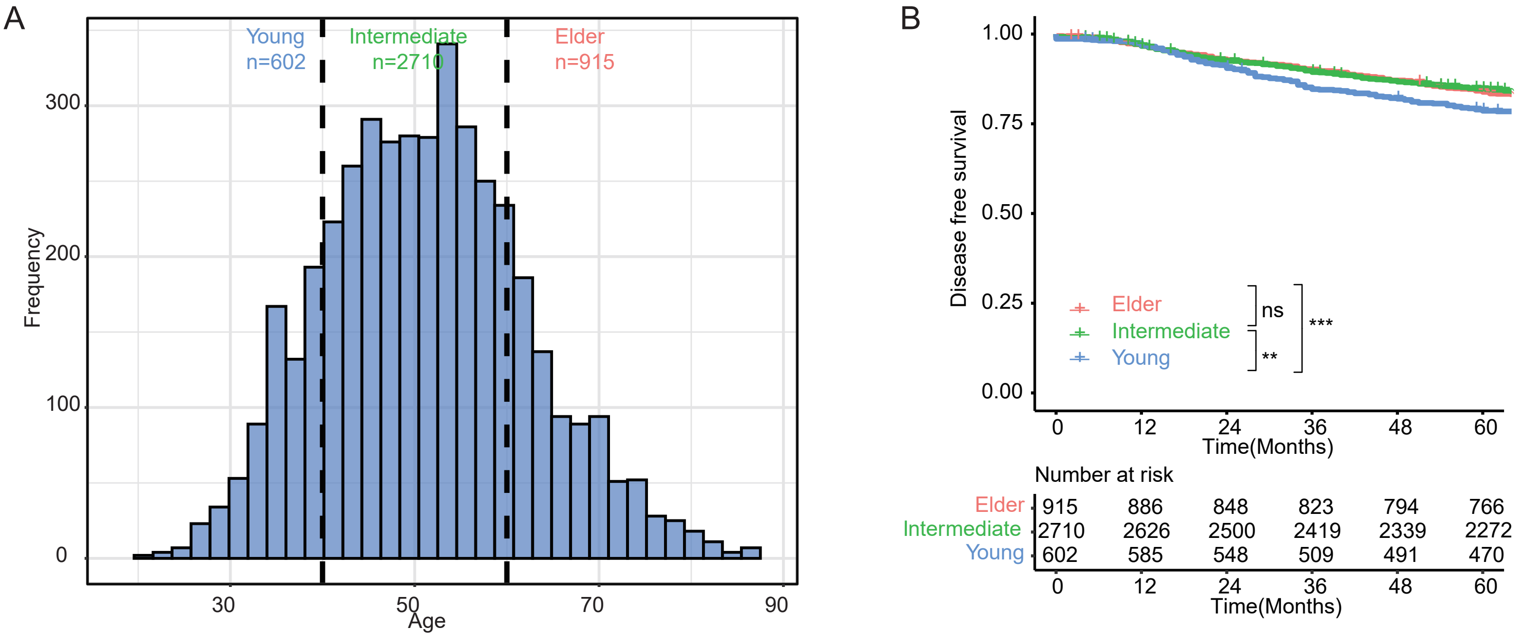

Supplementary Figure S1: Clinical features of breast cancer patients across different age groups (FUSCC).
(A) Distribution of age at diagnosis and the defined age groups in the FUSCC cohort.
(B) Disease free survival (DFS) in the FUSCC cohort. Survival comparisons were performed, and significant differences were annotated as **P < 0.01; ***P < 0.001; or not significant (ns) (P > 0.05).

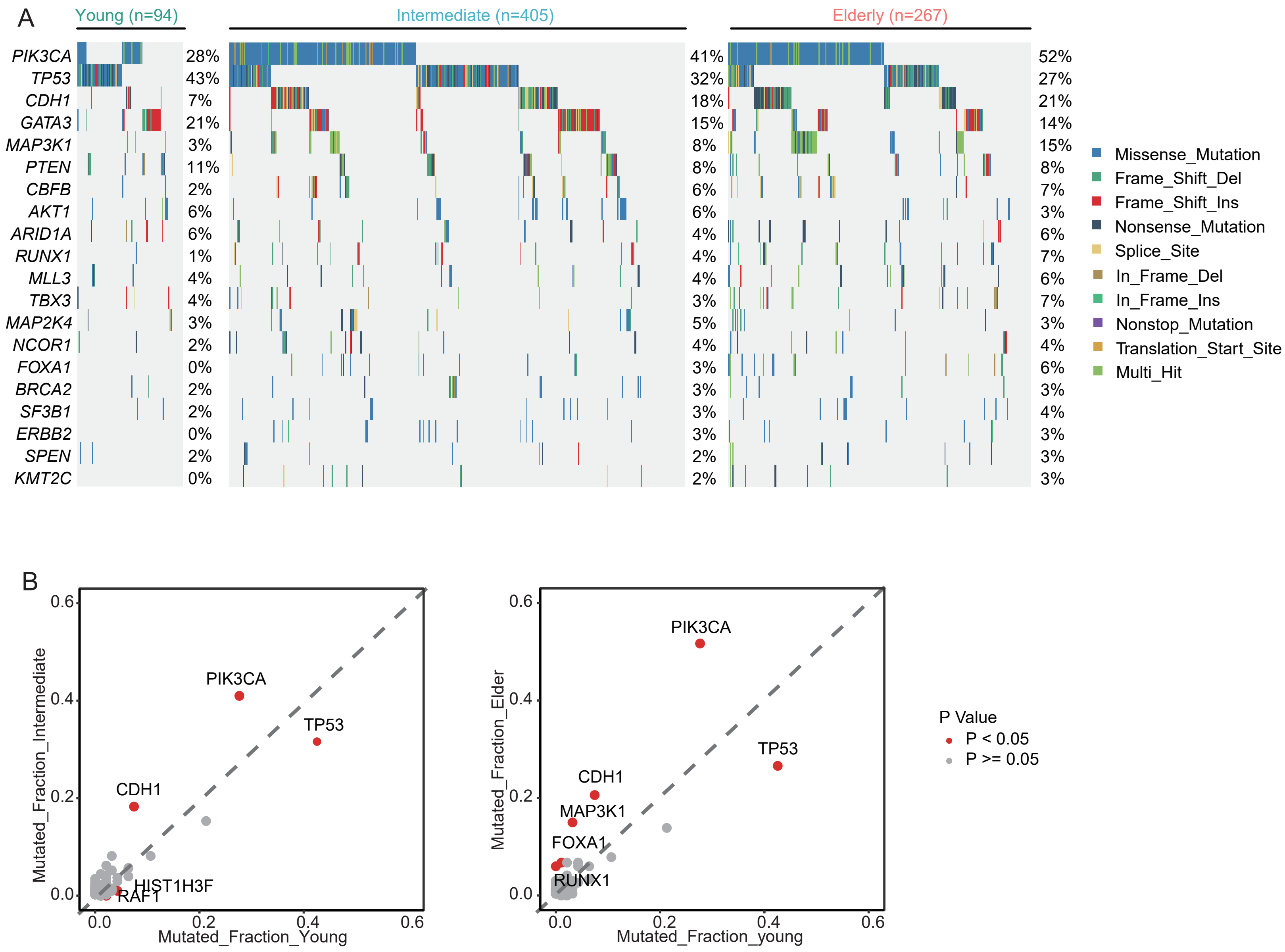

Supplementary Figure S2: Genomic features of breast cancer patients across different age groups (MSKCC). (A) Mutation profile of patients in elder, intermediate, and young groups. The top 20 genes were listed. (B) Comparison of mutations in young, intermediate (left), and elder (right) groups.

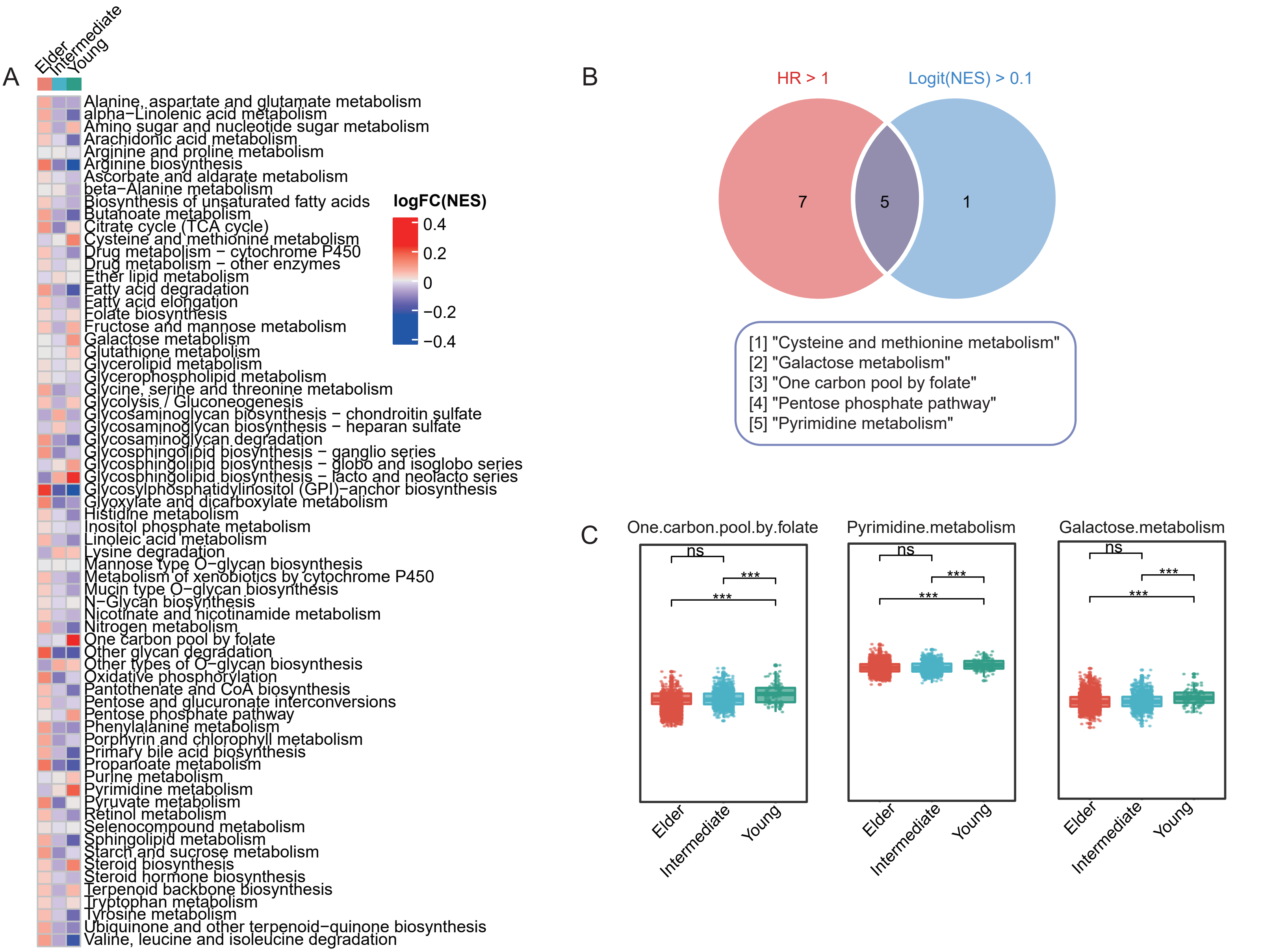

Supplementary Figure S3: Metabolic heterogeneity of breast cancer patients in different age groups (SCAN-B).

(A) Heatmap displaying the normalized enrichment score (NES) of 65 metabolic pathways in each age group.
(B) Venn diagram of the intersection between metabolic pathways upregulated in the young group (logit (NES) > 0.1) and pathways associated with worse disease-free survival (HR > 1).
(C) Box plots presenting NES values of three metabolic pathways in each age group; p-values: Kruskal-Wallis test for multiple comparisons and significant differences were annotated as ***P < 0.001 and ns P > 0.05.

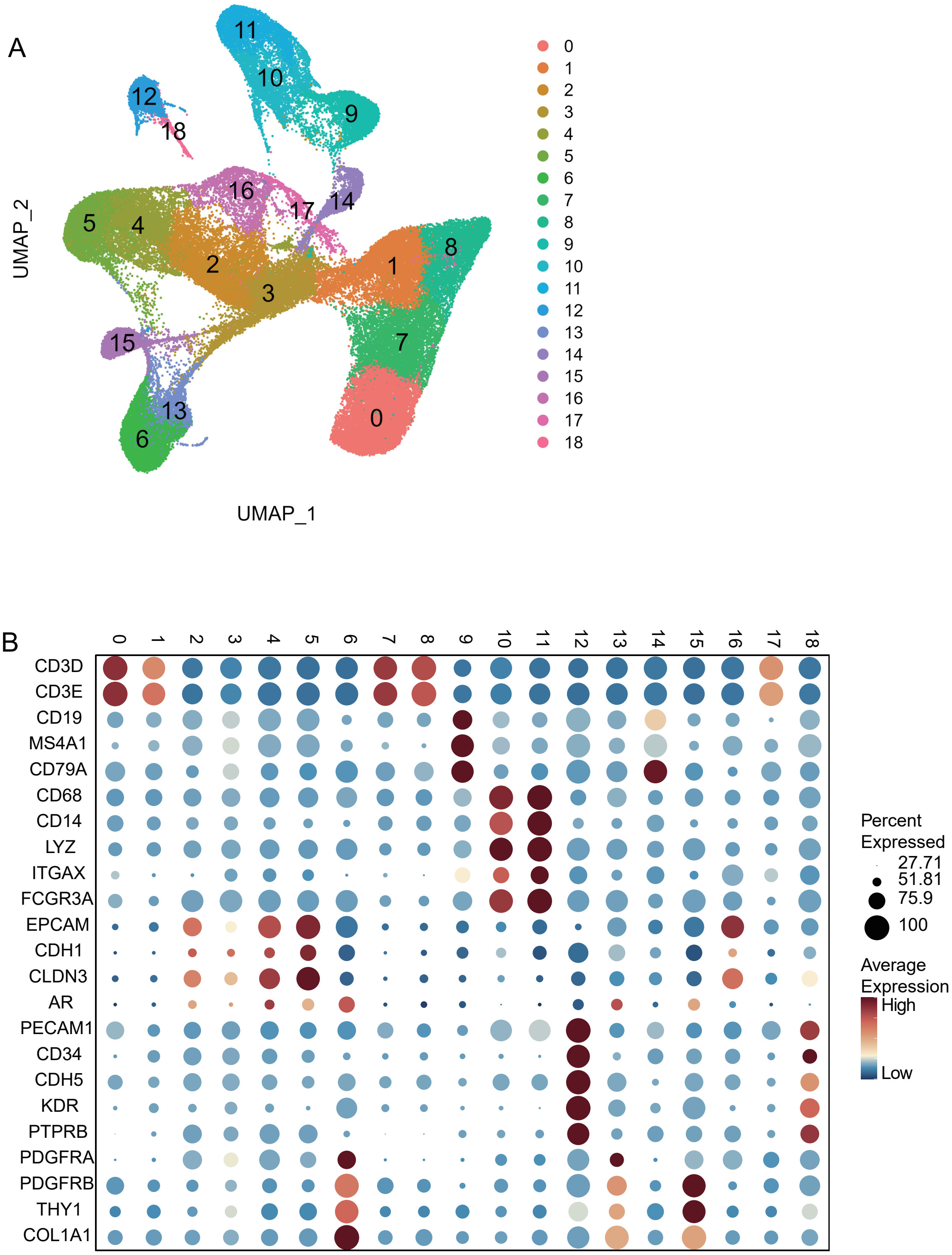

Supplementary Figure S4: Cellular composition of breast cancers and identification of cancer cells. (A) UMAP visualization of 84,854 cells by scRNA-seq and integrated across 35 primary breast tumors. (B) Dotplot visualizing averaged expression of cell markers across 19 clusters. T cells (CD3D, CD3E); B cells (CD19, MS4A1, CD79A); Epithelial cells (EpCAM, CDH1, CLDN3, AR); Endothelial cells (PECAM1).
